# Atypical Presentation of Disseminated Herpes Zoster as Abdominal Pain: A Case of Zoster-Associated Peritonitis in an Elderly Patient

**DOI:** 10.7759/cureus.79491

**Published:** 2025-02-23

**Authors:** Natsumi Yamamoto, Ryuichi Ohta, Akira Yamasaki

**Affiliations:** 1 Department of Community Care, Unnan City Hospital, Unnan, JPN; 2 Division of Respiratory Medicine and Rheumatology, Department of Multidisciplinary Internal Medicine, School of Medicine, Faculty of Medicine, Tottori University, Yonago, JPN

**Keywords:** abdominal pain, acyclovir, elderly patients, family medicine, general medicine, herpes zoster, peritonitis, postherpetic neuralgia, rural

## Abstract

Disseminated herpes zoster can present with a wide range of clinical manifestations, including visceral involvement. This case report describes an 89-year-old male who presented with severe abdominal pain, initially without any cutaneous lesions, leading to diagnostic challenges. The patient had acute-onset right lower abdominal pain that was persistent and worsened with movement. Initial diagnostic imaging revealed mild peritoneal edema over the ascending colon without evidence of bowel obstruction, vascular abnormalities, or free air. Due to the severity of the pain, symptomatic treatment was administered. Subsequently, a vesicular rash appeared on the right flank, corresponding to the pain site. This led to the diagnosis of disseminated herpes zoster with peritonitis. Antiviral therapy with intravenous acyclovir was initiated promptly, resulting in clinical improvement. However, postherpetic neuralgia (PHN) developed and required ongoing pain management. This case underscores the importance of including disseminated herpes zoster in the differential diagnosis of acute abdominal pain, particularly in elderly and immunocompromised patients. The absence of an initial rash can delay diagnosis, increasing the risk of complications. Early recognition and antiviral therapy prevent disease progression and improve patient outcomes. Moreover, PHN remains a significant concern, emphasizing the need for effective pain management and preventive strategies, including vaccination. Raising awareness among healthcare providers about atypical presentations of herpes zoster can facilitate early diagnosis and timely intervention, ultimately enhancing patient care.

## Introduction

Herpes zoster predominantly affects older adults, and its incidence is increasing due to the decline in both innate and acquired immunity [1]. In an aging society, herpes zoster has become a significant condition that adversely impacts the quality of life (QOL) and activities of daily living (ADL) of the elderly [2]. Most cases of herpes zoster present as unilateral rashes along a single dermatome, often accompanied by pain. This pain is a significant factor contributing to the decline in ADL [3].

On the other hand, disseminated herpes zoster, characterized by multi-dermatomal or bilateral involvement, occurs in older adults with chronic diseases or immunosuppressive conditions related to physical or psychological stress [4]. This condition is associated with higher infection rates and greater severity, making early detection and treatment critical [5].

Disseminated herpes zoster in older adults manifests diverse symptoms involving the skin and systemic areas innervated by affected nerves [6]. In typical cases, disseminated herpes zoster begins with rashes, progressing to complications such as meningitis, pleuritis, or peritonitis [6]. However, 5-15% of herpes zoster cases are atypical, presenting with pain preceding rash or with delayed rash onset. Such atypical presentations may also occur in disseminated herpes zoster [3].

We report a case of an elderly man who presented with acute right lower quadrant pain in the abdomen without an evident rash at the time of consultation, posing significant diagnostic challenges. This case highlights the importance of maintaining an appropriate differential diagnosis and considering the possibility of disseminated herpes zoster with delayed rash onset when evaluating severe pain that does not align with the clinical findings.

## Case presentation

An 89-year-old male presented to a rural community hospital with a chief complaint of severe pain extending from the epigastrium to the right flank. The patient had been living independently, but the pain had acutely developed one day prior. The pain was persistent with intermittent exacerbations, worsened by movement, and radiated throughout the abdomen. The severity of the patient's abdominal pain was assessed using the numerical rating scale, which was recorded as 10/10. He denied fever, chills, chest pain, vomiting, diarrhea, or dysuria. His past medical history included hypertension, chronic heart failure, atrial fibrillation, hypothyroidism, dyslipidemia, chronic prostatitis, benign prostatic hyperplasia, insomnia, and osteoarthritis. His medications included sacubitril/valsartan (200 mg), amlodipine (5 mg), bisoprolol (0.625 mg), pravastatin (10 mg), apixaban (5 mg), levothyroxine (25 μg), tadalafil (5 mg), and tamsulosin (0.2 mg) daily.

On presentation, his vital signs were as follows: Glasgow coma scale 15, temperature 35.6°C, blood pressure 161/77 mmHg, pulse 56 bpm, respiratory rate 20/min, and oxygen saturation (SpO₂) 96% on room air. Abdominal examination revealed tenderness from the epigastrium to the right lower quadrant, with positive tapping pain. Bowel sounds were normal. Right costovertebral angle (CVA) tenderness was noted. No cutaneous lesions were initially observed. Laboratory findings revealed mild anemia (Table 1).

**Table 1:** Initial laboratory data of the patient CRP: C-reactive protein; PT: prothrombin time; INR: international normalized ratio; APTT: activated partial thromboplastin time; eGFR: estimated glomerular filtration rate; Na: sodium; K: potassium; Cl: chloride; Ca: calcium

Marker	Level	Reference
White blood cells	5.9	3.5–9.1 × 10^3^/μL
Neutrophils	62.9	44.0–72.0%
Lymphocytes	22.8	18.0–59.0%
Hemoglobin	10.8	11.3–15.2 g/dL
Hematocrit	32.6	33.4–44.9%
Mean corpuscular volume	79.7	79.0–100.0 fl
Platelets	22.6	13.0–36.9 × 10^4^/μL
Total protein	7.1	6.5–8.3 g/dL
Albumin	4.0	3.8–5.3 g/dL
Total bilirubin	0.7	0.–1.2 mg/dL
Aspartate aminotransferase	20	8–38 IU/L
Alanine aminotransferase	18	4–43 IU/L
Lactate dehydrogenase	212	121–245 U/L
Amylase	51	44-132 IU/L
Lipase	25	7-60 IU/L
Blood urea nitrogen	16.3	8–20 mg/dL
Creatinine	0.72	0.40–1.10 mg/dL
eGFR	76.6	>60 ml/min/1.73m^2^
Creatine kinase	191	43-165 U/L
Serum Na	142	135–150 mEq/L
Serum K	3.9	3.5–5.3 mEq/L
Serum Cl	107	98–110 mEq/L
Serum Ca	9.29.5	8.8-10.2 mg/dL
CRP	0.00	<0.30 mg/dL
PT (%)	102.7	70-130 %
PT-INR	0.98	0.85-1.15
APTT (seconds)	29.2	25-40 seconds
Urine test	-	-
Leukocyte	Negative	Negative
Protein	Negative	Negative
Blood	Negative	Negative

White blood cell count, C-reactive protein, and liver function tests were within normal limits. Urinalysis showed no pyuria or bacteriuria.

**Figure 1:**
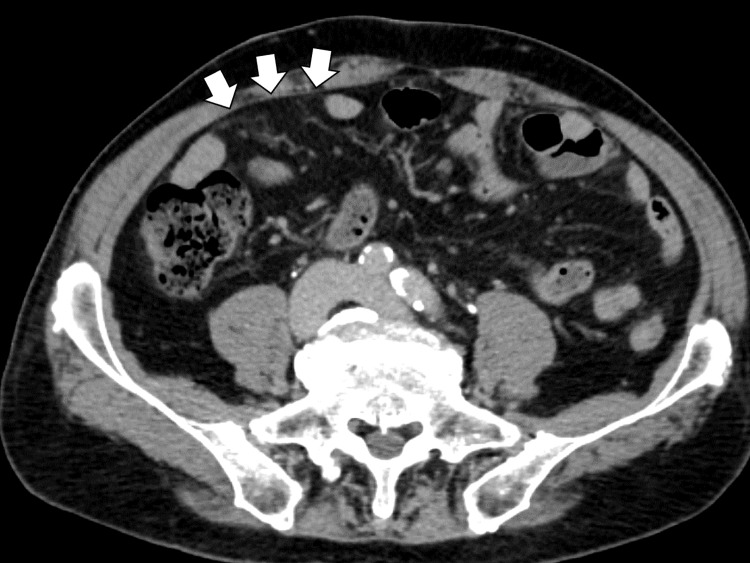
Abdominal computed tomography showing mild edema of the peritoneum over the anterior aspect of the ascending colon (white arrows).

Given the presentation of acute abdominal pain, differential diagnoses included bowel obstruction, gastrointestinal perforation, renal infarction, aortic dissection, and abdominal cutaneous nerve entrapment syndrome (ACNES). A contrast-enhanced CT of the chest to pelvis revealed no free air, bowel dilation, or obstruction. There was no evidence of vascular enlargement or renal perfusion defects. However, the CT showed mild edema of the peritoneum over the anterior aspect of the ascending colon, not specific for any disease (Figure 1).

**Figure 2:**
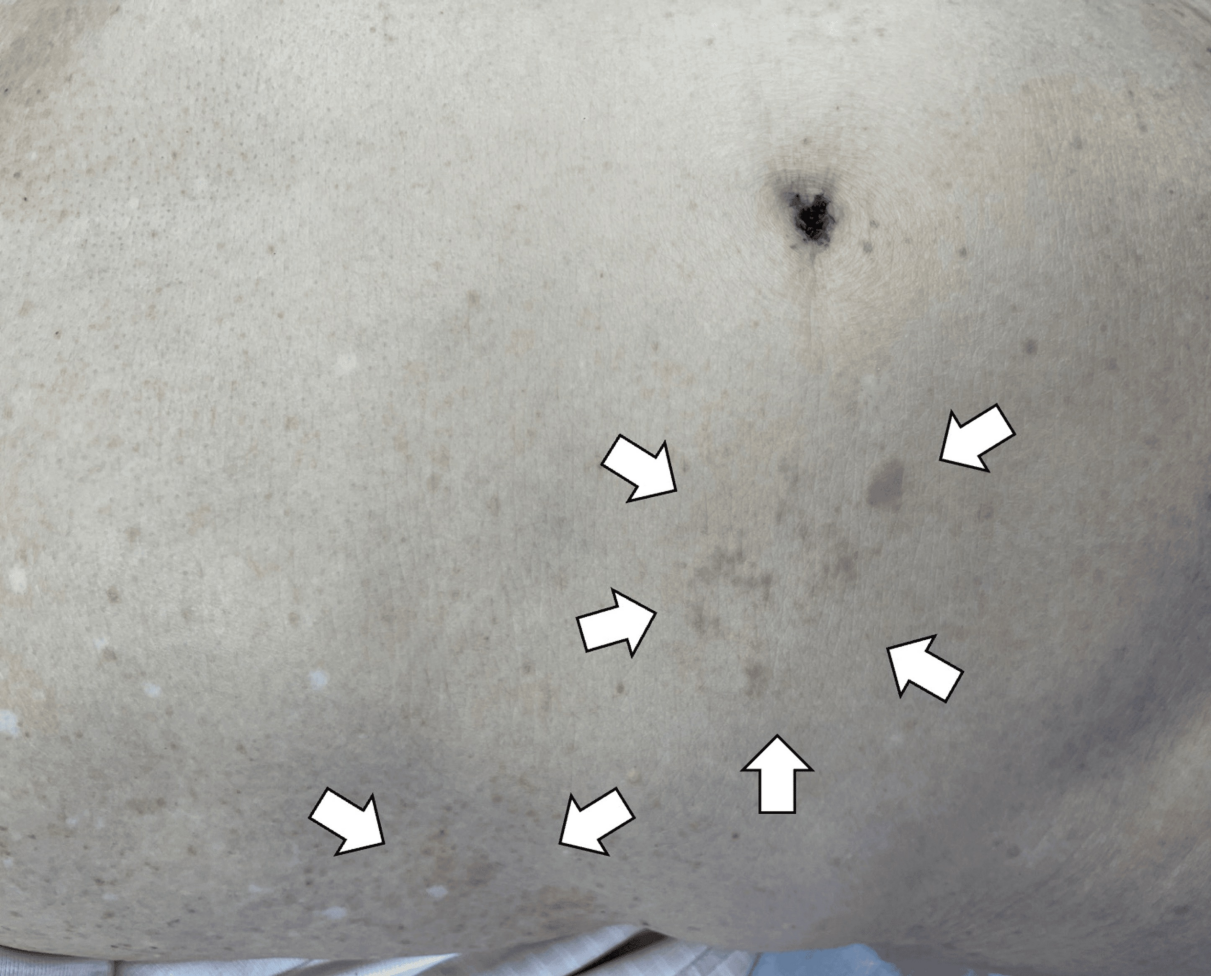
Abdominal rash extending along the right intercostal region to the lower back (white arrows).

Due to severe pain, acetaminophen (1000 mg IV) was administered, followed by diclofenac (60 mg suppository). Considering no specific findings of the CT scan and physical findings, we transiently diagnosed the patient with ACNES and prepared a trigger point injection. During the preparation, a vesicular rash emerged over the right flank, corresponding to the site of pain. The rash extended along the intercostal region to the lower back (Figure 2).

Based on these findings, the patient was diagnosed with disseminated herpes zoster with zoster-associated peritonitis.

**Figure 3:**
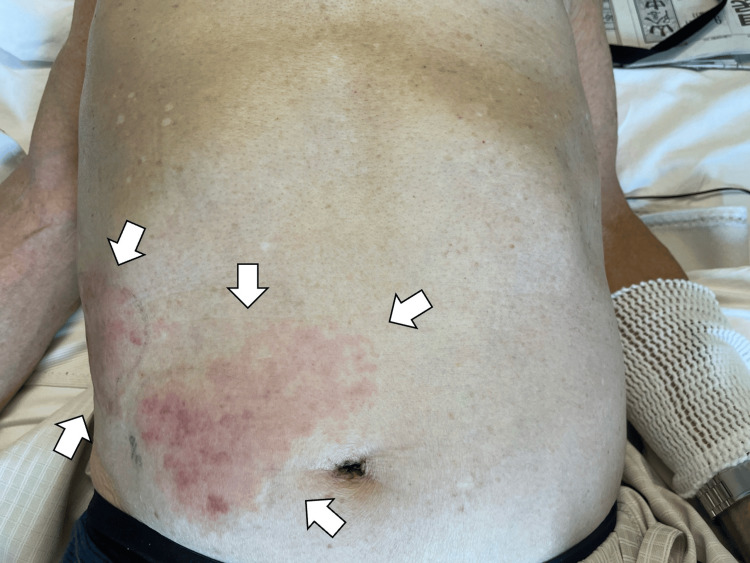
Vesicular lesions expanded over the right trunk (white arrows).

Intravenous acyclovir (500 mg three times daily) was initiated on the first day of hospitalization. Pain management included acetaminophen (1500 mg/day) and tramadol/acetaminophen combination therapy. By the third hospital day, vesicular lesions expanded over the right trunk, accompanied by worsening peritoneal and cutaneous pain (Figure 3).

Given the presence of neuropathic pain, pregabalin (75 mg/day) was started. A total of seven days of intravenous acyclovir was administered, leading to pain resolution, and the patient was discharged home. At an outpatient follow-up, the patient continued to experience postherpetic neuralgia (PHN), necessitating an increase in pregabalin to 300 mg/day alongside tramadol/acetaminophen combination therapy for symptom control.

## Discussion

This case is notable for presenting disseminated herpes zoster as peritonitis without an initially apparent rash at the first medical evaluation. This rare case suggests that disseminated herpes zoster can induce systemic symptoms without cutaneous manifestations. Disseminated herpes zoster is known to have a higher incidence in immunocompromised individuals and the elderly, with an increasing prevalence with age [7]. Although herpes zoster generally presents with a preceding rash, some cases have a delayed or absent rash, referred to as “zoster sine herpete" [8]. General physicians frequently encounter abdominal pain in elderly patients, and when symptoms of peritonitis are present despite inconclusive imaging findings, this condition should be considered as a potential diagnosis [9].

Including disseminated herpes zoster in the differential diagnosis of patients presenting with abdominal pain is particularly important for elderly and immunocompromised patients [10]. In this case, differentiating herpes zoster from conditions such as ACNES was initially challenging. However, the subsequent appearance of the rash allowed for an accurate diagnosis. Previous literature indicates that neurological symptoms preceding the rash in herpes zoster can often lead to misdiagnosis, emphasizing the importance of careful clinical observation [11,12]. Recently, the diagnosis of ACNES has increased in general practice, and trigger point treatments are sometimes performed diagnostically, but the potential for misdiagnosis has also been noted [13]. Physicians should maintain a broad differential diagnosis and be cautious about early diagnostic closure.

Furthermore, the timing of treatment initiation significantly impacts patient outcomes. Early administration of antiviral agents has been shown to reduce the severity of herpes zoster symptoms and prevent the progression of disseminated disease [14]. In this case, antiviral therapy was promptly initiated following the onset of the rash, leading to symptom resolution and preventing severe complications in the acute phase, thereby contributing to a favorable prognosis.

However, PHN remains a significant long-term challenge, substantially affecting patients’ quality of life. PHN is particularly common in elderly patients, and its prevention requires early treatment and adequate pain management [15]. In this case, pain control was managed with pregabalin and tramadol/acetaminophen combination therapy, but complete prevention of PHN was not achieved. PHN has been identified as a significant factor in reducing the quality of life in elderly patients [16]. In today’s aging society, not only early diagnosis and treatment of herpes zoster but also preventive strategies, including vaccination and further research into effective treatments for PHN, are necessary [2,17,18].

## Conclusions

This case highlights a rare presentation of disseminated herpes zoster manifesting as peritonitis without an initial rash. It underscores the necessity of including disseminated herpes zoster in the differential diagnosis of elderly or immunocompromised patients presenting with acute abdominal pain. General physicians should be aware of the possibility of delayed rash onset and ensure thorough observation and appropriate diagnostic investigations until a definitive diagnosis. Early initiation of antiviral therapy is crucial in preventing severe complications and improving patient outcomes. Postherpetic neuralgia remains a significant challenge, necessitating a comprehensive approach to pain management and prevention. This case reinforces the importance of awareness and education among healthcare professionals to enhance the diagnosis and treatment of disseminated herpes zoster, ultimately improving clinical practice and patient care.
